# Legionella pneumonia in Argentina: A disease cluster to worry about

**DOI:** 10.1016/j.amsu.2022.104969

**Published:** 2022-11-17

**Authors:** Yashendra Sethi, Nirja Kaka, Hitesh Chopra, Talha Bin Emran

**Affiliations:** Government Doon Medical College, Dehradun, Uttarakhand, India; GMERS Medical College Himmatnagar, Himmatnagar, Gujarat, India; Chitkara College of Pharmacy, Chitkara University, Punjab, India; Department of Pharmacy, BGC Trust University Bangladesh, Chittagong, 4381, Bangladesh; Department of Pharmacy, Faculty of Allied Health Sciences, Daffodil International University, Dhaka, 1207, Bangladesh

**Keywords:** Legionella, Pneumonia, COVID-19, Transmission, Signs and symptoms

Legionella is a facultative intracellular gram-negative coccobacillus with the reservoir being a common water body or aerosolized system. Although human-to-human transmission is uncommon it still presents as a public health hazard both due to the associated mortality and widespread transmission through a common source [1,2]. Legionella classically causes atypical pneumonia (Legionnaires' disease). The common signs and symptoms include but are not limited to fever, chills, headache, hyponatremia, diarrhea, relative bradycardia, and altered mental status. A third of patients also present with blood-streaked phlegm or hemoptysis. It is also known to cause a milder Pontiac fever (flu-like symptoms without pneumonia) in immunocompetent patients. Despite these distinct features, it may often be misdiagnosed, and if left untreated, has a mortality of 30–50% [1]. The incubation period for Legionnaires’ disease is usually 2–10 days, however, up to 16 days have been reported [3].

The incidence of Legionnaires disease varies greatly with the level of surveillance and reporting. The USA, Europe, and Australia report 10–15 cases per million population each year with 75–80% of reported cases being over 50 years and around 60–70% of them males [3]. Despite the less reported human-to-human transmission, in a world opening up with the resumption of global travel and gatherings, Legionella-contaminated aerosols can be generated in the moist environment of commercial heating, ventilation and air conditioning (HVAC) systems used extensively [4].

The recent clustering of 11 cases at Tucuman, Argentina reported by The Pan American Health Organization (PAHO) and the ministry of health, Argentina makes the discussion about legionella, its transmission, and prevention imperative. Although the exact information for the outbreak is still being looked into, the *L. pneumophila*, being the most common species to be associated with outbreaks seems to be the culprit with the possible transmission source being inhalation of contaminated aerosols [5].

With the recent pandemic of Coronavirus disease 2019 (COVID-19), the literature has grown to underline the expanded role of ‘ferritin’ in iron metabolism. It is a positive acute phase reactant that gets elevated in many infections including the disease caused by severe acute respiratory syndrome coronavirus 2 (SARS-CoV-2). It is well known that iron acquisition is essential for the growth of and survival of legionella bacteria [2]. The hyperferritinemia seen during SARS-CoV-2 infection and in the post-COVID phase can form a conducive environment for several iron-dependent pathogenic organisms like mucormycosis [6], and legionella, especially with the threat of newer and re-emerging variants.

The diagnosis of the disease depends on clinical suspicion and lab tests including culture, urine antigen tests, polymerase chain reaction (PCR), direct fluorescent antibody (DFA), and serology (Table 1) [7]. The mild cases without pneumonia usually recover without a pharmacological intervention whereas the moderate to severe Legionnaires' disease requires prompt treatment focused on antibiotic therapy. The most potent agents are fluoroquinolones (levofloxacin>moxifloxacin) and macrolides [8]. But, with the propensity to produce outbreaks, the role of preventive and public health strategies cannot be overstated.

**Table 1:** Diagnostic tests for Legionella [7].

Diagnostic test	Sensitivity (%)	Specificity (%)	Advantages	Disadvantages
Culture	20–80	100	• High specificity	• Difficult
			• Can detect all species	• Slow (>5 days)
			• Best evidence	• BYCE agar (not easily available)
Urinary antigen test (UAT)	70–100	95–100	• Rapid	• Just available for *L. pneumophila* serogroup 1 (most common)
Polymerase Chain Reaction (PCR)	95–99	>99	• Rapid	• Limited availability
			• Can be done even on pathological samples	
			• Wide serogroup detection	
Direct Fluorescent Antibody (DFA) test	25–75	>95	• Rapid	• Technically difficult
			• Can be done even on pathological samples	• Reagents may be difficult to obtain
			• Wide serogroup detection	
Serology	80–90	>99	• Can be done even on pathological samples	• Limited time window for testing
			• Wide serogroup detection	• Tough titer based discrimination

The preventive efforts revolve around containing the contamination of reservoirs and restricting the creation of aerosols [3]. This can be done by promoting regular maintenance and disinfection of cooling towers, maintaining strict HVAC disinfection, reducing stagnation of water, and maintaining adequate biocide levels in spa pools. We advocate the enactment of appropriate public health measures to curb the spread of disease and promote preparedness in form of timely disease cluster identification, enhanced disease reporting, focused monitoring and surveillance, and environmental screening. It is important for the world of tomorrow that the lessons from the previous outbreaks must not just be used to improve mitigative response but also aggrandize preventive strategies.

## Ethical approval

This article does not require any human/animal subjects to acquire such approval.

## Source of funding

This study received no specific grant from any funding agency in the public, commercial, or not-for-profit sectors.

## Author contribution

**Yashendra Sethi:** Conceptualization, Data curation, Writing-Original draft preparation, Writing- Reviewing and Editing. **Navneet Kaur:** Conceptualization, Data curation, Writing-Original draft preparation, Writing- Reviewing and Editing. **Hitesh Chopra:** Data curation, Writing-Original draft preparation, Writing- Reviewing and Editing. **Talha Bin Emran:** Conceptualization, Writing-Reviewing and Editing, Visualization.

## Research registration number

1. Name of the registry: Not applicable.
2. Unique Identifying number or registration ID:Not applicable.
3. Hyperlink to your specific registration (must be publicly accessible and will be checked): Not applicable.

## Guarantor

Talha Bin Emran, Ph.D., Associate Professor, Department of Pharmacy, BGC Trust University Bangladesh, Chittagong 4381, Bangladesh. T: +88-030-3356193, Fax: +88-031-2550224, Cell: +88-01819-942214. https://orcid.org/0000-0003-3188-2272. E-mail: talhabmb@bgctub.ac.bd.

## Consent

Not applicable.

## Declaration of competing interest

We have read and understood the policy on declaration of interests and have no relevant interests to declare. The responsibility for the content lies with the author and the views stated herein should not be taken to represent those of any organisations or groups with and for which he works.

## References

[1] Tkatch L.S., Kusne S., Irish W.D., Krystofiak S., Wing E. (1998). Epidemiology of Legionella pneumonia and factors associated with Legionella-related mortality at a tertiary care center. Clin. Infect. Dis..

[2] Cianciotto N.P. (2015). An update on iron acquisition by Legionella pneumophila: new pathways for siderophore uptake and ferric iron reduction. Future Microbiol..

[3] Legionellosis (n.d. (2022). https://www.who.int/news-room/fact-sheets/detail/legionellosis.

[4] Cunha B.A., Burillo A., Bouza E. (2016). The Lancet.

[5] (2022). Update – Legionella identified as cause of cluster of pneumonia cases in Tucuman. Argentina - PAHO/WHO | Pan American Health Organization.

[6] Bhadania S., Bhalodiya N., Sethi Y., Kaka N., Mishra S., Patel N., Wasim A.U., Joshi S.S., Shah K. (2021). Hyperferritinemia and the extent of mucormycosis in COVID-19 patients. Cureus.

[7] (2022). Legionnaires disease diagnosis, treatment, and prevention. CDC.

[8] Jasper A.S., Musuuza J.S., Tischendorf J.S., Stevens V.W., Gamage S.D., Osman F., Safdar N. (2021). Are fluoroquinolones or macrolides better for treating Legionella pneumonia? A systematic review and meta-analysis. Clin. Infect. Dis..

